# Progression pattern of myopic maculopathy according to the severity of diffuse chorioretinal atrophy and choroidal thickness

**DOI:** 10.1038/s41598-022-07172-w

**Published:** 2022-02-23

**Authors:** Un Chul Park, Eun Kyoung Lee, Chang Ki Yoon, Baek-Lok Oh

**Affiliations:** Department of Ophthalmology, Seoul National University College of Medicine, 103 Daehak-ro, Jongno-gu, Seoul, 110-799 Korea

**Keywords:** Retinal diseases, Macular degeneration

## Abstract

This observational cohort study aimed to evaluate the progression pattern of diffuse chorioretinal atrophy (DCA) according to its severity. Highly myopic eyes with DCA were graded according to its extent in the 532-nm (green) and 633-nm (red) wavelengths images of the Optos ultra-widefield scanning laser ophthalmoscope at baseline: grade 1 and 2 were defined when increased reflectance at peripapillary region, not beyond the fovea, were observed in red laser image only and in both laser images, respectively; grade 3 and 4 were defined when increased reflectance beyond the fovea were observed in red laser image only and in both laser images, respectively. A total of 307 eyes (221 patients) were included, and progression of myopic maculopathy during follow-up of ≥ 3 years was evaluated. The mean visual acuity and subfoveal choroidal thickness (CT) differed among DCA grades (*P* = 0.015 and *P* < 0.001); a higher DCA grade had worse visual acuity and thinner choroid. During follow-up, development of patchy atrophy (PA) was observed in 3.2%, 5.5%, 12.8%, and 23.2% (*P* < 0.001), while changes in lacquer crack (LC) and/or development of myopic macular neovascularization were observed in 20.6%, 29.1%, 33.3%, and 15.8% (*P* = 0.061) of 63, 110, 39, and 95 eyes with DCA grade of 1, 2, 3, and 4 at baseline, respectively. New LC formation tended to occur in eyes with thicker CT at baseline compared to PA development and progression of pre-existing LC. In highly myopic eyes with DCA, progression pattern of myopic maculopathy is different according to its severity and CT at baseline. Grading based on separated wavelength images of ultra-widefield scanning laser ophthalmoscope is useful to evaluate the severity and prognosis of DCA in Asian patients with high myopia.

## Introduction

High myopia is one of the major causes of irreversible visual impairment worldwide, particularly in East Asia where myopia is highly prevalent^1–4^. Visual impairment in highly myopic eyes mainly results from myopic maculopathy, which refers to a range of atrophic, tractional, and neovascular changes in the posterior pole^5^. Among theses, chorioretinal atrophic change is most commonly observed in highly myopic eyes, and the current classification system for myopic maculopathy, the Meta-analysis of Pathologic Myopia (META-PM) Study Group classification^6^, is mainly based on atrophic components as follows: tessellated fundus only (category 1), diffuse chorioretinal atrophy (category 2), patchy chorioretinal atrophy (category 3), and macular atrophy (category 4).

Diffuse chorioretinal atrophy (DCA), category 2, is characterized by an ill-defined yellowish lesion in the posterior pole in highly myopic eyes without evidence of a patchy atrophic (PA) lesion, which is a well-defined gray-white lesion resulting from an absence of retinal pigment epithelium (RPE) and choriocapillaris. The DCA may be subclassified into peripapillary and macular DCA according to the extent of atrophic changes^7,8^. Choroidal thinning plays a key role in the development and progression of DCA^9^; it begins nasal to the fovea and progresses toward the fovea, finally involving the entire posterior pole. However, influence of DCA severity on the myopic maculopathy progression is unknown, and a detailed grading scheme of DCA which can simply reflect its severity and prognosis has not been reported yet.

The Optos ultra-widefield retinal imaging is a scanning laser ophthalmoscope that obtains images using two wavelengths laser scan (green, 532 nm and red, 633 nm). Separated images by two different wavelengths can provide further information according to the depth of penetration and have been reported to be useful in some diseases involving the choroid^10–12^. In the same way, in highly myopic eyes, separated ultra-widefield images of two wavelengths of laser could help evaluate the severity of DCA, reflecting the extent of choroidal thinning and associated changes in the posterior pole. In addition, a recent study based on optical coherence tomography (OCT) revealed that nasal and subfoveal choroidal thicknesses (CTs) are useful to diagnose and differentiate peripapillary and macular DCA, suggesting relevant cut-off values for each category^9^. In this study, we evaluated clinical features and progression pattern of DCA according to its severity based on the separated red and green laser scans of ultra-widefield scanning laser ophthalmoscope and the CT measured using spectral domain OCT.

## Methods

In this retrospective observational study, the medical records of patients with high myopia examined by ultra-widefield retinal imaging at the Retina Center of the Seoul National University Hospital between January 2015 and December 2017 and were reviewed. High myopia was defined as an axial length of ≥ 26.5 mm or a myopic refractive error ≤—6.0 diopters. Based on the baseline ultra-widefield retinal images, myopic maculopathy was classified into four categories according to the META-PM Study Group classification^6^. Among the patients with category 2 myopic maculopathy, i.e. DCA, those who were followed-up for at least 36 months based on ultra-widefield retinal imaging were included in the analysis. The exclusion criteria were as follows: (1) poor image quality of ultra-widefield image; (2) other retinal disorders, such as diabetic retinopathy, retinal vascular diseases, age-related macular degeneration, and uveitis; (3) active myopic macular neovascularization (MNV); (4) previous history of photodynamic therapy, and anti-vascular endothelial growth factor (VEGF) treatment; and (5) history of vitreoretinal surgery before or during the study period. The protocol and study design were approved by the Institutional Review Board of the Seoul National University Hospital (IRB no. 2003–231-1115), which waived the informed consent due to retrospective nature of this study, and this study was conducted in accordance with the tenets of the Declaration of Helsinki.

All patients underwent a comprehensive ophthalmic examination, including best-corrected visual acuity (BCVA), refractive error (spherical equivalent), slit lamp examination, fundus examination, axial length measurement, ultra-widefield retinal imaging, and spectral domain OCT. For statistical analysis, BCVA was converted to the logarithm of minimal angle of resolution (logMAR) scale. In the analysis of refractive error, eyes with previous history of cataract surgery or refractive surgery were excluded. The axial length measurement was performed with ocular biometry (IOLMaster 500; Carl Zeiss Meditec, Jena, Germany). The ultra-widefield retinal image was taken with an Optos 200Tx scanning laser ophthalmoscope (Optos PLC, Scotland, UK) centered on the macula by a skilled technician under the same condition to minimize the variability in the images. Based on the B-scan images of spectral domain OCT (Cirrus HD-OCT, Carl Zeiss Meditec, Dublin, CA) including the fovea, CT was measured vertically from the outer edge of the hyperreflective line of the RPE to the choroid-scleral junction at the subfovea and 3,000 μm nasal to the fovea. Considering the influence of long axial length on the dimension on the OCT image, the actual point of nasal CT measurement was adjusted based on a previously reported formula: *t* = 3.382 × 0.01306 × (axial length − 1.82) × *s*, where *t* and *s* represent the actual dimensions and measurements on the OCT image, respectively^13^.

**Figure 1:**
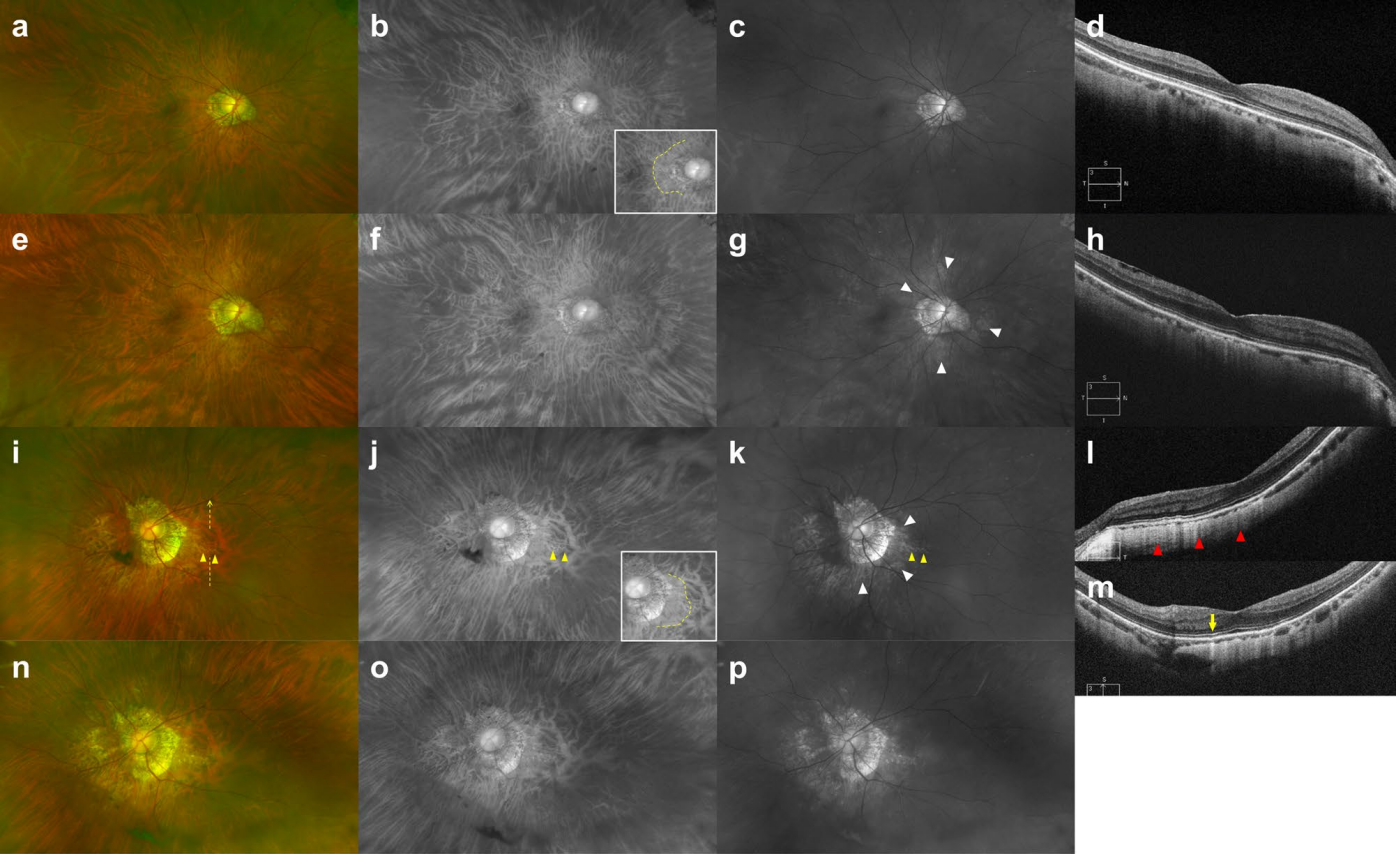
Pseudocolor (**a,e,i,n**), red separation (**b,f,j,o**), and green separation (**c,g,k,p**) images by the ultra-widefield scanning laser ophthalmoscope and spectral domain OCT (**d,h,l,m**) scan images traversing the fovea in highly myopic eyes with diffuse chorioretinal atrophy (DCA) grades 1 and 2. **(a–d)** At baseline examination of a 62-year-old man with DCA grade 1, area of increased reflectance was limited to the temporal peripapillary region not beyond the fovea in the red separation image (yellow dotted line in **b** inlet, decreased tessellation due to blurring of choroidal vessels), but no reflectance was observed in the green separation image. **(e–h)** Fifty-two months later, slightly increased reflectance at the peripapillary region was observed in the green separation image (white arrowheads). **(i–m)** At baseline examination of a 59-year-old woman with DCA grade 2, areas of increased reflectance in the red separation image (yellow dotted line in **j** inlet, decreased tessellation due to blurring of choroidal vessels) and green separation image (white arrows) were limited to the temporal peripapillary region not beyond the fovea. A horizontal lacquer crack, which appeared as yellowish linear lesion temporal to the optic disc (yellow arrowheads), was observed more clearly in the green separation image. Note much thinner choroid and increased light penetration nasal to the fovea compared to the region temporal to the fovea in horizontal OCT image (**l**, red arrowheads) and discontinuity of RPE and an increased light penetration at the lacquer crack in vertical OCT image (**m**, yellow arrow). A yellow dotted arrow in **i** indicates the vertical OCT scan line (**m**). A middle third was interrupted not to obscure underlying findings. **(n–p)** Forty months later, enlargement of preexisting lacquer crack was observed.

**Figure 2:**
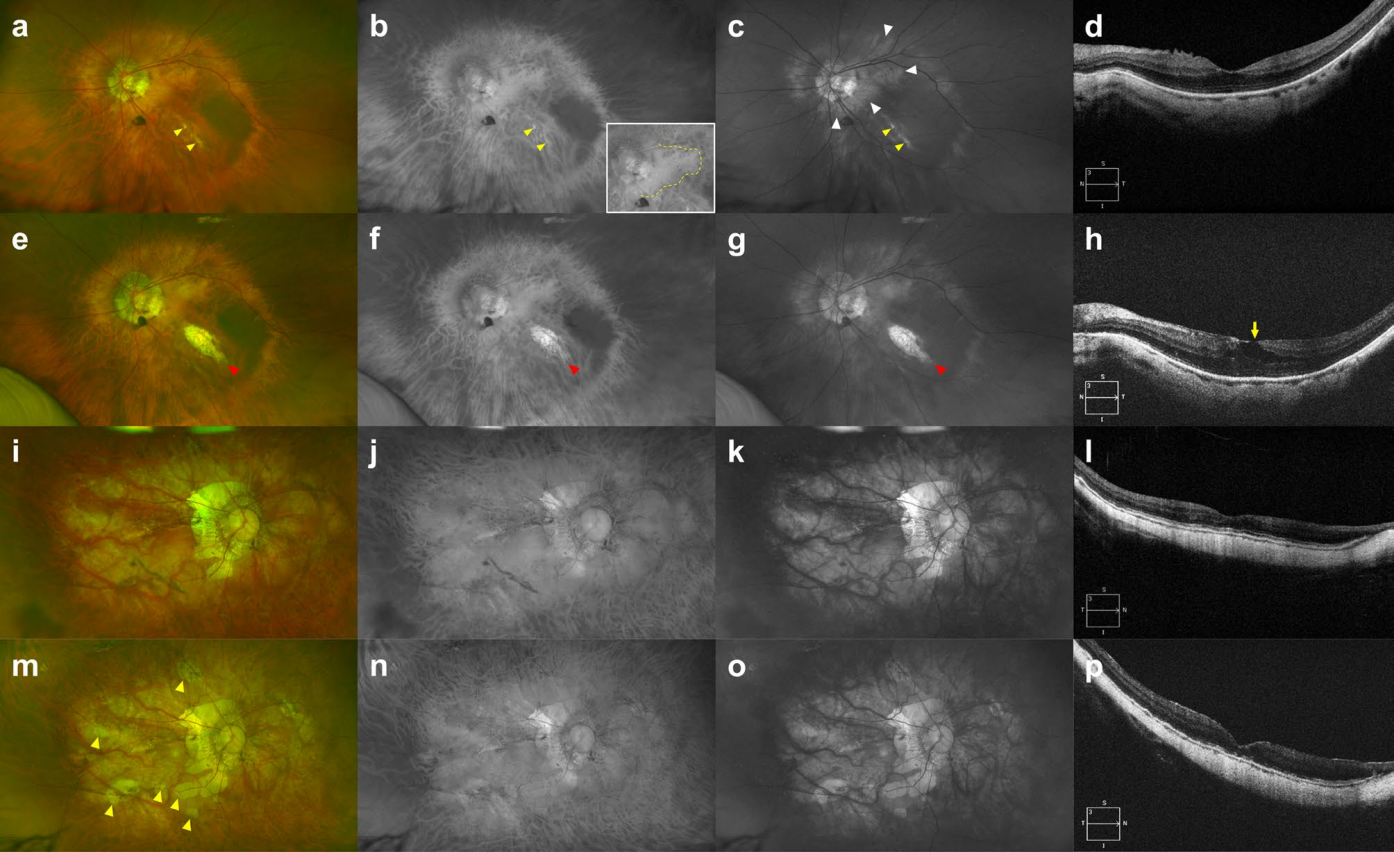
Pseudocolor (**a,e,i,m**), red separation (**b,f,j,n**), and green separation (**c,g,k,o**) images by the ultra-widefield scanning laser ophthalmoscope and spectral domain OCT (**d,h,l,p**) horizontal scan images traversing the fovea in highly myopic eyes with diffuse chorioretinal atrophy (DCA) grades 3 and 4. **(a–d)** At baseline examination of a 73-year-old woman with DCA grade 3, area of increased reflectance was beyond the fovea in the red separation image (yellow dotted line in **b** inlet, decreased tessellation due to blurring of choroidal vessels), but area of increased reflectance in the green separation image was limited to the temporal peripapillary region not beyond the fovea (white arrowheads). An oblique lacquer crack, which appeared as yellowish linear lesion (yellow arrowheads), was observed more clearly in the green separation image. **(e–h)** Sixty-seven months later, patchy atrophic lesion developed at the preexisting lacquer crack at baseline (red arrowheads). Note the development of thin epiretinal membrane and foveal retinoschisis in OCT image (yellow arrow). **(i–l)** At baseline examination of a 55-year-old woman with DCA grade 4, areas of increased reflectance were observed beyond the fovea over the entire posterior fundus both in the red separation image (decreased tessellation due to blurring of choroidal vessels) and green separation image. **(m–p)** Sixty-four months later, development of multiple patchy atrophic lesions was observed (yellow arrowheads). Note extremely thin choroid both at baseline and final OCT images.

The DCA was classified into four grades according to the extent of increased reflectance at the peripapillary and macular region in the red and green separation images. Brightness and contrast of images were not adjusted during the DCA grading. In the red separation image, “area of increased reflectance” was defined as the region with decreased tessellation due to blurring of choroidal vessels compared to the surrounding definite tessellations. In the green separation image, it was defined as the region with any visible reflectance with or without tessellation. For Grade 1, increased reflectance in the red separation image was limited to the temporal peripapillary region not beyond the fovea, but green separation image showed no increased reflectance. Grade 2 represented increased reflectance were limited to the temporal peripapillary region not beyond the fovea both in the red and green separation images. For grade 3, area of increased reflectance in the red separation image was extended beyond the fovea, but increased reflectance in the green separation image was not observed or limited to the temporal peripapillary region not beyond the fovea. Grade 4 represented increased reflectance were observed beyond the fovea, usually in the entire posterior fundus, both in the red and green separation images. Representative images of ultra-widefield scanning laser ophthalmoscope and OCT in patients with each DCA grade are shown in Figs. 1 and 2. The presence of lacquer crack (LC) at baseline and its progression or new development during follow-up were recorded. A lacquer crack was observed as fine and irregular yellowish linear lesion in and around macula. When located in the area of DCA, it had a brighter color against the background atrophic changes, which was more contrasted in the red or green separation images (Supplement Fig. S1). Posterior staphyloma was determined to be present when abnormal pigmentary changes in the pseudo-color image or abnormal reflectance in the red or green separation image indicating the staphyloma border were observed^14,15^.

Clinical and ocular characteristics were compared among the four DCA grade groups using one-way analysis of variance or chi-square test as appropriate. In each eye, myopic maculopathy was regarded as progressed when the following findings were observed during follow-up: (1) development of a new patchy atrophic lesion; (2) enlargement of DCA lesion; (3) development of a new LC or enlargement of the preexisting LC; and (4) development of myopic MNV. Progression patterns of (1) and (2) were regarded as the progression of myopic atrophic maculopathy and (3) and (4) as the progression of myopic neovascular maculopathy according to a recently suggested ATN classification^5^. Red and green separation images of ultra-widefield scanning laser ophthalmoscope were assessed by two independent retinal specialists (EKL and CKY) for DCA grade at baseline and myopic maculopathy progression during follow-up, and any discrepancies were adjudicated by a senior retinal specialist (UCP). For all parameters, interobserver agreement was ≥ 90% and kappa coefficient was ≥ 0.80. The CT measurements of two graders were averaged for statistical analysis. All statistical analyses were performed using SPSS software version 22.0 (SPSS Inc., Chicago, IL), and *P* values of < 0.05 were considered statistically significant.

## Results

Of the 591 highly myopic patients who underwent ultra-widefield retinal imaging during the study period and had an available follow-up ultra-widefield retinal image 36 months or more, 378 eyes of 253 patients were determined as having DCA at baseline. All patients were Korean. Among these, 71 eyes were excluded: 14 eyes due to poor quality of images, 17 due to the presence of other chorioretinal disorders, 17 due to a history of intravitreal anti-VEGF injection to treat myopic MNV, and 23 due to a history of vitreoretinal surgery before or during the study period. In total, 307 eyes of 221 patients were included in the analysis. The mean age was 59.8 ± 12.9 years (range, 19–86), and mean axial length was 29.8 ± 2.6 mm (range, 24.7–36.6). According to the extent of chorioretinal atrophic changes in the red and green separation images, 63 (20.5%), 110 (35.8%), 39 (12.7%), and 95 (30.9%) eyes were classified as having DCA of grade 1, 2, 3, and 4, respectively.

Baseline demographic and ocular characteristics of the study eyes are listed in Table 1. There were significant differences among the grades of DCA in age, axial length, refractive error, subfoveal and nasal CT, and BCVA in logMAR scale; eyes with a higher grade of DCA tended to have an older age, longer axial length, greater myopic refractive error, thinner choroid, and worse BCVA. Nasal CT of < 56.5 μm, which were recently reported as optimal OCT-based diagnostic criteria for peripapillary DCA^9^, was met by 97.7% of entire eyes. Subfoveal CT of < 62 μm, the criteria for macular DCA, was met by 94.8% of eyes with DCA grade ≥ 3. Eyes with a higher grade DCA were more likely to have a higher frequency of LC and posterior staphyloma.

**Table 1:** Baseline characteristics and Demographics of patients according to the baseline grades of diffuse chorioretinal atrophy.

Parameters	Total	DCA Grade 1	DCA Grade 2	DCA Grade 3	DCA Grade 4	*P*-value
Number of eyes (%)/patients	307 eyes (100.0%)/221 patients	63 eyes (26.6%)	110 eyes (35.8%)	39 eyes (12.7%)	95 eyes (30.9%)	–
Mean age, years (range)	59.8 ± 12.9 (19–86)	56.6 ± 14.6 (19–80)	61.0 ± 12.7 (21–86)	63.2 ± 10.8 (20–85)	61.3 ± 11.1 (26–82)	0.011
Gender, number of eyes (%) of women	234 eyes (83.5%)/169 women	45 eyes (71.4%)	84 eyes (76.4%)	29 eyes (74.4%)	76 eyes (80.0%)	0.654
Mean axial length, mm (range)	29.8 ± 2.6 (24.7–36.6)	27.8 ± 2.5 (24.7–33.5)	29.0 ± 2.4 (25.7–35.8)	29.9 ± 2.1 (25.3–32.6)	31.5 ± 1.9 (25.1–36.6)	< 0.001
Mean refractive error, diopter (range)*	−12.6 ± 5.2 (−4.0–−26.0)	−9.8 ± 3.9 (−4.8–−18.0)	−11.8 ± 5.0 (−4.0–−26.0)	−14.0 ± 5.8 (−4.5–−25.5)	−15.4 ± 4.9 (−4.9–−23.8)	< 0.001
Mean baseline BCVA, logMAR (range, Snellen equivalent)	0.43 ± 0.41 (−0.08–2.50, 20/54)	0.31 ± 0.34 (−0.08–1.70, 20/41)	0.42 ± 0.38 (−0.08–1.70, 20/53)	0.52 ± 0.42 (−0.08–2.00, 20/66)	0.50 ± 0.46 (−0.08–2.50, 20/63)	0.015
Choroidal thickness						
Mean nasal choroidal thickness, μm	24.8 ± 14.4 (0–83)	31.1 ± 14.1 (0–73)	27.5 ± 14.1 (0–83)	19.7 ± 12.7 (0–55)	19.5 ± 13.3 (0–63)	< 0.001
Nasal choroidal thickness < 56.5 μm^†^	300 (97.7%)	60 (95.2%)	107 (97.3%)	39 (100%)	94 (98.9%)	
Mean subfoveal choroidal thickness, μm	41.1 ± 27.4 (7–171)	58.7 ± 38.1 (9–172)	43.3 ± 25.4 (13–123)	38.0 ± 18.9 (13–98)	28.2 ± 14.6 (7–69)	< 0.001
Subfoveal choroidal thickness < 62 μm^†^	254 (82.7%)	40 (63.5%)	87 (79.1%)	36(92.3%)	91 (95.8%)	
Presence of lacquer crack	115 (37.5%)	14 (22.2%)	31 (28.2%)	20 (51.3%)	50 (52.6%)	< 0.001
Presence of posterior staphyloma	188 (61.2%)	28 (44.4%)	58 (52.7%)	25 (64.1%)	77 (81.1%)	< 0.001
Follow-up period, months (range)	48.5 ± 11.1 (36–71)	48.6 ± 10.6 (36–70)	47.5 ± 11.5 (36–71)	51.1 ± 12.5 (36–69)	48.5 ± 10.5 (36–68)	0.384

DCA = Diffuse chorioretinal atrophy; BCVA = best-corrected visual acuity; logMAR = logarithm of minimal angle of resolution.

*Phakic eyes without history of refractive surgery.

^†^Optimal cut-off values of nasal choroidal thickness (< 56.5 μm) to predict the presence of peripapillary DCA and subfoveal choroidal thickness (< 62 μm) to predict the presence of macular DCA suggested in an optical coherence tomography-based study^9^.

**Figure 3:**
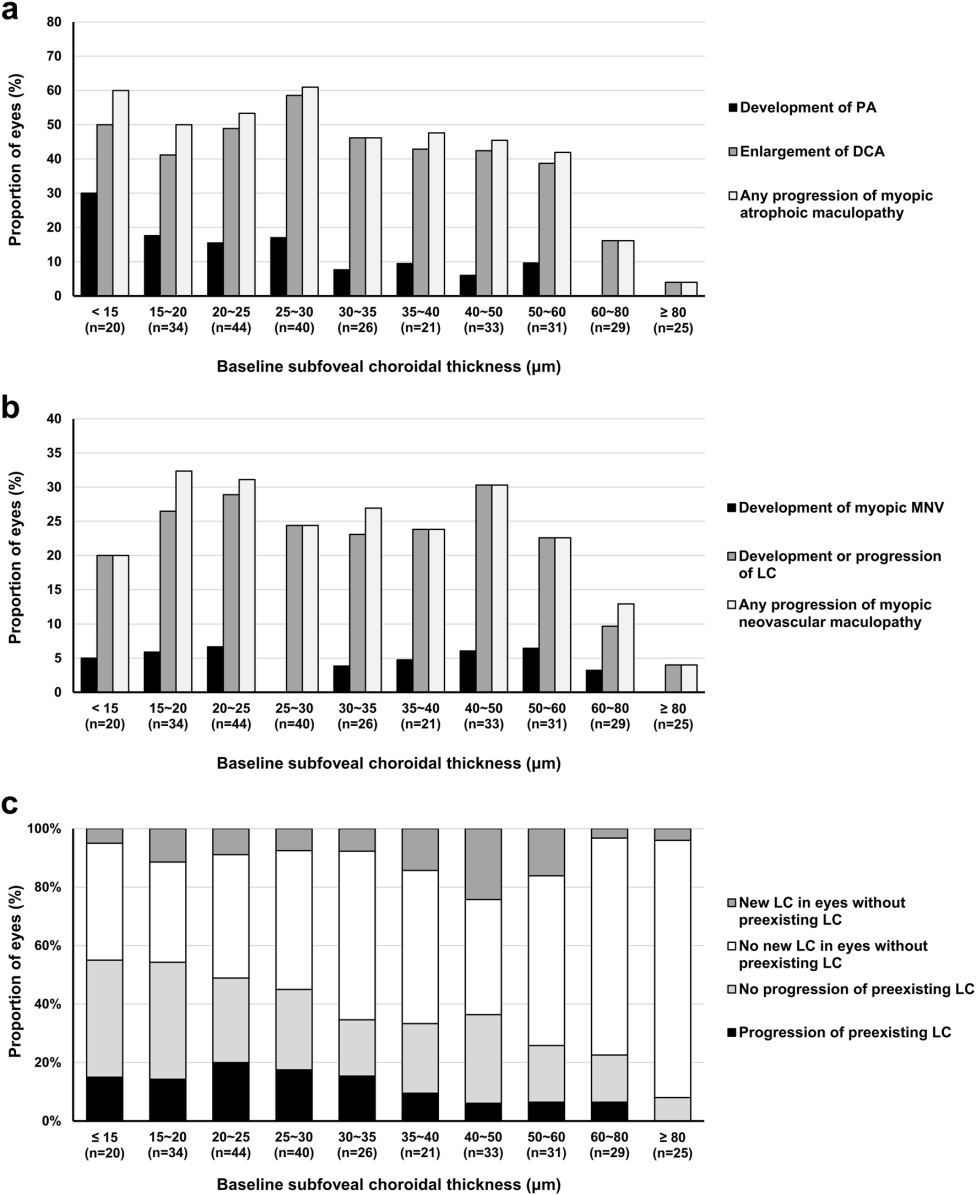
Frequency of myopic atrophic maculopathy progression during follow-up **(a)**, myopic neovascular maculopathy progression during follow-up **(b)**, and prevalence of LC at baseline and change of LC during follow-up **(c)** according to the baseline subfoveal choroidal thickness. PA = patchy atrophy; DCA = diffuse chorioretinal atrophy; myopic MNV = myopic macular neovascularization; LC = lacquer crack.

The mean follow-up period was 48.5 ± 11.1 months (range, 36–71) and did not differ among the eyes of DCA grade of 1 to 4 (*P* = 0.384). The progression pattern according to the baseline DCA grade is shown in Table 2. During follow-up, both enlargement of DCA and development of PA were observed more frequently in eyes with higher DCA grades (*P* < 0.001 for both). Development of PA was observed in 2 of 63 eyes (3.2%), 6 of 110 eyes (5.5%), 5 of 39 eyes (12.8%), and 22 of 95 eyes (23.2%) with DCA grade of 1, 2, 3, and 4, respectively. In 35 eyes with PA development during follow-up, PA lesions in 21 eyes (60.0%) were originated from DCA. In those eyes, DCA was grade 4 in 18 eyes (85.7%) at baseline and in all 21 eyes (100%) at the final visit, and new PA lesions were within the area of DCA both in the red and green separation images. Development of PA lesion was associated with preexisting or new LC in 13 eyes (37.1%) or scar formation of myopic MNV in one eye (2.9%). Compared to eyes without PA development during follow-up, those with PA development had significantly lower subfoveal CT (27.9 ± 14.2 μm vs. 42.8 ± 28.3 μm; *P* = 0.002) and nasal CT (18.1 ± 15.5 μm vs. 25.6 ± 14.1 μm; *P* = 0.003). The frequency of myopic atrophic maculopathy progression during follow-up, including DCA enlargement and PA development, according to the baseline subfoveal CT, are shown in Fig. 3A. Eyes with lower subfoveal CT tended to have a higher risk of PA development, while the frequency of DCA enlargement was comparable among the eyes with subfoveal CT < 60 μm.

**Table 2:** Progression pattern according to the baseline grade of diffuse chorioretinal atrophy.

Parameters	DCA Grade 1 (63 eyes)	DCA Grade 2 (110 eyes)	DCA Grade 3 (39 eyes)	DCA Grade 4 (95 eyes)	*P*-value
Enlargement of DCA	11 eyes (17.5%)	33 eyes (30.0%)	25 eyes (64.1%)	54 eyes (56.8%)	< 0.001
Development of patchy atrophy	2 eyes (3.2%)	6 eyes (5.5%)	5 eyes (12.8%)	22 eyes (23.2%)	< 0.001
Any progression of myopic atrophic maculopathy*	13 eyes (20.6%)	35 eyes (31.8%)	26 eyes (66.7%)	60 eyes (63.2%)	< 0.001
Changes in lacquer crack	13 eyes (20.6%)	29 eyes (26.4%)	12 eyes (30.8%)	14 eyes (14.7%)	0.115
Development of new lacquer crack^†^	6 / 49 (12.2%)	12 / 79 (15.2%)	3 / 19 (15.8%)	2 / 45 (4.4%)	0.326
Progression of preexisting lacquer crack^‡^	7 / 14 (50.0%)	17 / 31 (54.8%)	9 / 20 (45.0%)	12 / 50 (24.0%)	0.029
Development of myopic macular neovascularization	2 eyes (3.2%)	8 eyes (7.3%)	2 eyes (5.1%)	1 eye (1.1%)	0.139
Any progression of myopic neovascular maculopathy**	13 eyes (20.6%)	32 eyes (29.1%)	13 eyes (33.3%)	15 eyes (15.8%)	0.061

DCA = Diffuse chorioretinal atrophy.

*Includes enlargement of DCA and development of patchy atrophy lesion.

^†^In eyes without lacquer crack at baseline.

^‡^In eyes with preexisting lacquer crack at baseline.

**Includes changes in lacquer crack and development of myopic macular neovascularization.

Development of new LC in eyes without LC at baseline and progression of LC in eyes with preexisting LC tended to be less frequent in eyes with DCA grade 4 than in those with grade ≤ 3 (Table 2). However, statistically significant difference according to the DCA grades was observed only for the progression of preexisting LC (*P* = 0.029). In 23 eyes with new development of LC during follow-up, 14 eyes (60.9%) developed LC out of DCA lesion. Development of myopic MNV was most frequent in eyes with DCA grade 2 (8 of 110 eyes; 7.3%) and least frequent in eyes with DCA grade 4 (1 of 95 eyes; 1.1%) but was not different among the DCA grades (*P* = 0.139). The frequency of myopic neovascular maculopathy progression during follow-up, including the progression of preexisting LC, development of new LC, and development of myopic MNV, according to the baseline subfoveal CT, are shown in Fig. 3B and were similar across the eyes with baseline subfoveal CT < 60 μm. Prevalence of LC at baseline and change of LC during follow-up, including development of new LC or progression of preexisting LC, are detailed in Fig. 3C. Prevalence of LC increased gradually with the decrease in the baseline subfoveal CT. Development of new LC in eyes without preexisting LC was most frequent in eyes with baseline subfoveal CT between 40 and 50 μm. In comparison, the progression of preexisting LC had a tendency to occur at lower subfoveal CT at baseline.

## Discussion

This observational cohort study, which included highly myopic eyes with DCA followed-up for longer than three years, shows that the progression pattern of each component of myopic maculopathy, including PA development, DCA enlargement, LC changes, and myopic MNV development, was different according to the DCA severity and choroidal thinning at baseline. In this study, DCA was graded based on the extent of atrophic change on the separated ultra-widefield images by two different wavelength lasers. The grading scheme showed good correlation with axial length, BCVA, and CT, suggesting that it can be adopted as a useful and easy severity scale for DCA at least in Asian patients with high myopia.

In this study, development of a new PA lesion or enlargement of DCA were more likely to occur in eyes with advanced DCA. In particular, all new PA lesions not originated from LC and myopic MNV developed in eyes that reached grade 4 DCA at final visit, within the regions of atrophoic change. This indicates that majority of PA lesion is very likely to develop in highly myopic eyes with DCA change over the entire posterior pole beyond the fovea and severe choroidal thinning, which is thought to be the most advanced form of DCA. On the contrary, progression of neovascular components of myopic maculopathy, which includes changes in LC and myopic MNV according to a recently suggested ATN classification^5^, tended to occur more frequently in eyes with DCA grade of ≤ 3, although statistical significance was only observed for the progression of pre-existing LC. The combined incidence of these changes during follow-up was similar across the baseline subfoveal CT. However, as shown in the Fig. 3C, development of new LC in eyes without preexisting LC tended to occur at greater subfoveal CTs at baseline (most frequently at 40–50 μm) compared to the progression of preexisting LC (most frequently at 20–25 μm) or development of PA (most frequently at < 15 μm).

The reason why PA lesion develops at a more advanced stage of DCA with thinner choroid while new formation of LC and myopic MNV is observed at earlier stage of DCA with less thinned choroid is unclear. One possibility is that the linear Bruch membrane defect, namely LC, may result from incipient mechanical stress exerted on the posterior pole during the early axial elongation of highly myopic eyes and can develop even outside the area with DCA change. Meanwhile, in eyes with more advanced DCA and extremely thin choroid, a prolonged decrease in choroidal perfusion may increase the vulnerability and risk of hole formation in the Bruch membrane to develop a new PA lesion, but myopic MNV might be less likely to develop due to lower VEGF level following decreased choroidal vascularity. Further studies are required to elucidate the mechanisms of two different types of Bruch membrane defects in highly myopic eyes with DCA.

The progression rate of myopic maculopathy in highly myopic eyes with DCA, the META-PM category 2, was reported to be various from 14.2 to 70.8%^8,16–21^, probably due to differences in the study population and length of follow-up. Although most studies have detailed progression patterns, e.g. enlargement of DCA and development of PA, myopic MNV, and LC during follow-up, no study investigated progression pattern according to the DCA severity or CT at baseline. In a hospital-based study by Fang et al.^8^ that evaluated the progression of myopic maculopathy, development of PA lesion was observed in 28.6%, and the development of myopic MNV or new LC was in 12.4% among 217 eyes with DCA at baseline during a mean follow-up of 18 years. These were greater than but in a similar ratio to the progression rates of 11.4% and 7.5% for each pattern in the present study with a relatively short mean follow-up period. However, in a recent prospective study by Li et al.^17^, which included much younger highly myopic patients with a mean age of 21.5 years, the ratio between the development of PA and LC was reversed, being observed in 4.5% and 19.3% of eyes with DCA for each pattern during a 4-year follow-up. Considering the progressive nature of myopic maculopathy^4^, younger patients in the study by Li et al. are likely to have less severe atrophic changes compared to ours and Fang et al.’s study, and the results seem to support our hypothesis that PA is more likely to develop during the more advanced stage of DCA compared to LC and myopic MNV.

In addition to a much wider field of view covering up to 200° of the fundus, the Optos ultra-widefield scanning laser ophthalmoscope is advantageous over the conventional photograph because separating two wavelength laser scan can provide further information according to their penetration depths^22^. Normally, a red laser with 633 nm penetrates deeper into the retina and choroid, better visualizing the choroidal vessels. A green laser with 532 nm cannot penetrate deeper than the RPE due to shorter wavelength and provides better images of the retinal surface and vessels. However, changes in the choroidal status may increase the penetration of each laser, resulting in the visualization of a deeper layer. For example, in eyes with chronic stage of Vogt-Koyanagi-Harada disease with sunset glow fundus, which is characterized by choroidal thinning and depigmentation, severe blurring is observed in the red laser image due to increased reflectance from the inner sclera, while choroidal vessels are better visualized in the green laser image due to depigmentation and atrophic change of RPE^12^.

Due to the thinned choroid and retina in highly myopic eyes, lasers of both wavelengths may penetrate deeper compared to non-myopic eyes. Choroidal thinning in highly myopic eyes occurs initially at the peripapillary region nasal to the fovea and enlarges toward and beyond the fovea^7,19,23^. Owing to this progressive nature of choroidal thinning, in this study, the severity of DCA could be graded based on the extent of atrophic change in the separated wavelength images. Over the area of DCA, choroidal thinning is revealed as whitish blurring in the red laser image. In an area with more severe choroidal thinning and secondary changes in the retina and RPE, penetration of green laser, which is not deeper than RPE in normal eyes, may be also enhanced leading to whitish blurring also in the green laser image. This grading system well represents the severity of DCA because it shows gradual differences in axial length, BCVA, and frequency of posterior staphyloma or LC according to the DCA grades. More importantly, an increase in the DCA grade was associated with gradual choroidal thinning, which was revealed to play an important role in the progression from no myopic maculopathy to DCA in a recent OCT-based study^9^.

In addition to its retrospective nature, there are some limitations to this study. First, this study was performed only in Korean patients. Penetration depths of lasers could be different among races according to the basal level of fundus pigmentation, and it is unclear whether the results of this study could be applied for races other than East Asian. Second, the influence of other aspects of myopic maculopathy, such as dome-shaped macula and myopic traction maculopathy, were not considered in the analysis. Third, this study had a relatively short follow-up period of ≥ 3 years to reveal the long-term progression pattern of DCA more precisely. Fourth, based on the results of this study, it cannot be concluded which eyes are at risk of myopic neovascular maculopathy development during the early stage of DCA, while others only show progression of atrophic changes without neovascular progression. Fifth, usefulness of combining separated wavelength image findings in the detection of LC, which may have increased the prevalence of LC compared to the previous studies^9^, has not been validated, and further study is required. However, mechanical break of RPE-Bruch membrane-choriocapillaris complex in LC lesions may more increase the reflectance from inner sclera^24,25^, making LCs more prominent against background atrophic changes in the green separation image. Lastly, presence of posterior staphyloma could be more clearly determined using widefield OCT showing gradual thinning of choroid toward its edge^26^, but this image modality was not included in this study.

In conclusion, in highly myopic eyes with DCA, the development of PA lesions is more likely to occur at a more advanced stage of DCA and thinner choroid compared to LC change and myopic MNV formation. The grading system of DCA based on the separated wavelength images of ultra-widefield scanning laser ophthalmoscope showed good correlation with the CT and could be useful imaging tool to assess the severity DCA and to predict the progression pattern of myopic maculopathy during follow-up in Asian patients with high myopia.

## Supplementary Information

Supplementary Information.

## Data Availability

The datasets generated during and/or analyzed during the current study are available from the corresponding author on reasonable request.
