# Supplementary material for: Progression pattern of myopic maculopathy according to the severity of diffuse chorioretinal atrophy and choroidal thickness
Source: Sci Rep. 2022 Feb 23;12:3099. doi: 10.1038/s41598-022-07172-w (PMC8866404; doi:10.1038/s41598-022-07172-w)
Supplement: Supplementary file 1 — Supplementary Information. [file 41598_2022_7172_MOESM1_ESM.pdf]

Title: Progression pattern of myopic maculopathy according to the severity of diffuse chorioretinal atrophy and choroidal thickness

Authors: Un Chul Park, MD, PhD[1], Eun Kyoung Lee, MD, PhD[1], Chang Ki Yoon, MD[1], Baek-Lok Oh, MD[1]

[1]Department of Ophthalmology, Seoul National University College of Medicine, Seoul, Korea

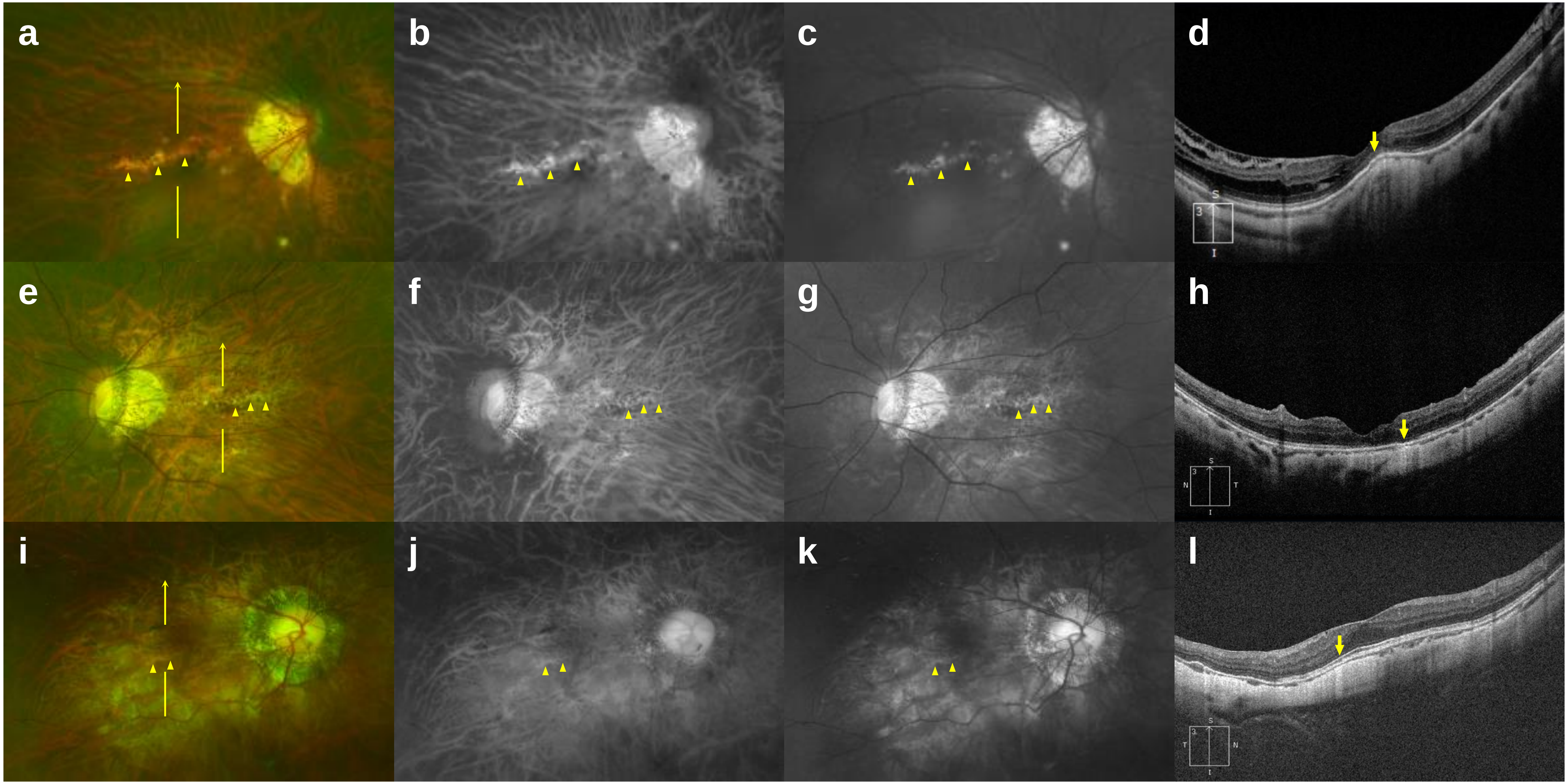

Supplement Figure S1., Pseudo-color (**a, e, I**), red separation (**b, f, j**), and green separation (**c, g, k**) images by the ultra-widefield scanning laser ophthalmoscope and spectral domain OCT (**d, h, l**) vertical scan images in highly myopic eyes with lacquer cracks (yellow arrowheads). **(a-d)** In an eye with mild diffuse atrophy, lacquer crack as a yellowish linear lesion is easily visible. In green separation image, increased reflectance is observed at only lacquer crack lesion. **(e-l)** In two eyes with severe diffuse atrophy, lacquer cracks are observed as linear lesions with brighter color against the background atrophic changes, which was more contrasted in the separated wavelength images, especially the green separation image. Vertical OCT images show discontinuities in RPE and increased light penetrance at the lacquer crack lesion (yellow arrows). Yellow lines in pseudo-color images indicate the direction of OCT scan, and a middle third was interrupted not to obscure underlying findings.
